# Crystal structure of *Caulobacter crescentus* polynucleotide phosphorylase reveals a mechanism of RNA substrate channelling and RNA degradosome assembly

**DOI:** 10.1098/rsob.120028

**Published:** 2012-04

**Authors:** Steven W. Hardwick, Tobias Gubbey, Isabelle Hug, Urs Jenal, Ben F. Luisi

**Affiliations:** 1 Department of Biochemistry, University of Cambridge, Tennis Court Road, Cambridge CB2 1GA, UK; 2 Biozentrum, University of Basel, Klingelbergstrasse 50/70, 4056 Basel, Switzerland

**Keywords:** polynucleotide phosphorylase, RNA degradosome, *Caulobacter crescentus*, RNA–protein interactions, molecular ratchet, conformational asymmetry

## Abstract

Polynucleotide phosphorylase (PNPase) is an exoribonuclease that cleaves single-stranded RNA substrates with 3′–5′ directionality and processive behaviour. Its ring-like, trimeric architecture creates a central channel where phosphorolytic active sites reside. One face of the ring is decorated with RNA-binding K-homology (KH) and S1 domains, but exactly how these domains help to direct the 3′ end of single-stranded RNA substrates towards the active sites is an unsolved puzzle. Insight into this process is provided by our crystal structures of RNA-bound and apo *Caulobacter crescentus* PNPase. In the RNA-free form, the S1 domains adopt a ‘splayed’ conformation that may facilitate capture of RNA substrates. In the RNA-bound structure, the three KH domains collectively close upon the RNA and direct the 3′ end towards a constricted aperture at the entrance of the central channel. The KH domains make non-equivalent interactions with the RNA, and there is a marked asymmetry within the catalytic core of the enzyme. On the basis of these data, we propose that structural non-equivalence, induced upon RNA binding, helps to channel substrate to the active sites through mechanical ratcheting. Structural and biochemical analyses also reveal the basis for PNPase association with RNase E in the multi-enzyme RNA degradosome assembly of the α-proteobacteria.

## 2. Introduction

The phosphorolytic exoribonuclease polynucleotide phosphorylase (PNPase; EC 2.7.7.8) is encoded in the genomes of bacteria and eukaryotes, and in those domains of life the ribonuclease plays multifaceted roles in regulation and environmental response [1,2]. PNPase of pathogenic bacteria influences complex phenotypes associated with infection, such as tissue-invasive virulence in *Salmonella enterica* [3,4] and host-effector secretion in *Yersinia* sp. [5]. In *Escherichia coli*, PNPase contributes to the decay of bulk RNA, quality control of ribosomal RNA, cold shock response, and the turnover of small regulatory RNA [6–11]. More recently, PNPase has been shown to play a role in stabilizing some small regulatory RNA species in *E. coli* [12]. *Deinococcus radiodurans* PNPase is implicated in ribosomal RNA decay as part of the response to starvation conditions [13]. Human PNPase contributes to global changes in gene expression associated with terminal differentiation [14] and to the transport of RNA into the mitochondrion [15]. In the chloroplast, PNPase is involved in phosphate homeostasis [16].

**Figure 1.**
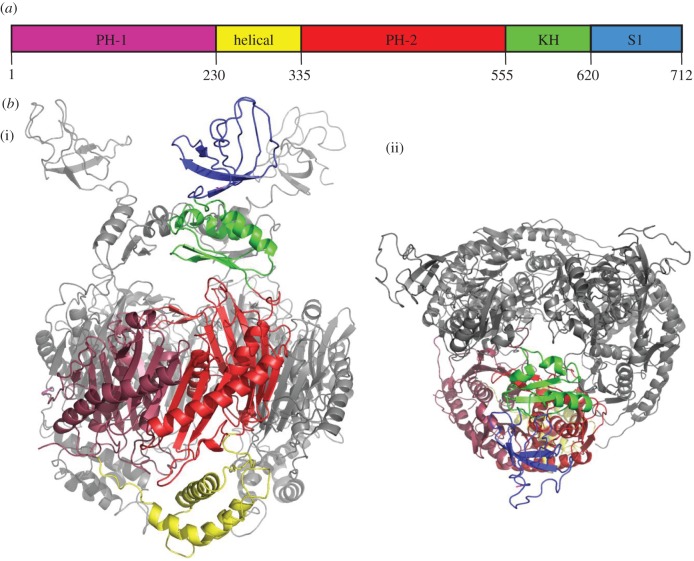
The structure of *Caulobacter crescentus* polynucleotide phosphorylase. (*a*) Linear schematic of domain organization, showing the two RNase PH domains, the helical domain, the KH and S1 RNA-binding domains. (*b*) A schematic of the structure of the trimeric *C. crescentus* PNPase. (i) Side view and (ii) perpendicular view along the threefold axis. For one of the protomers, the individual subdomains are colour coded according to the scheme in figure 1*a*. The other two protomers are grey.

All known PNPase homologues share the same modular organization of conserved structural domains, depicted schematically in figure 1*a*. Two domains, both resembling closely the phosphorolytic exoribonuclease RNase PH, almost certainly have originated from duplication and fusion of an ancestral gene. While the C-terminal RNase PH-like domain catalyses phosphorolytic attack of RNA, the N-terminal domain has lost this capacity; instead, it contributes to the ring-like quaternary structure of the trimeric PNPase assembly [17]. Two recurrent structural motifs that are found in many RNA-binding proteins, the S1 and K-homology (KH) domains, are appended at the C-terminus of PNPase [18]. Finally, an all-helical domain is situated between the two RNase PH-like domains. The crystal structures of *Streptomyces antibioticus*, *E. coli* and human PNPase reveal how these domains are spatially organized [17,19]. Foremost, the structures reveal that the trimer is stabilized mainly by interactions of neighbouring RNase PH-like domains, and the quaternary structure generates a central channel coincident with the molecular threefold axis. The exosome assemblies of eukaryotes and archaea share molecular ancestry with PNPase, and have a similar ring-like architecture of RNase PH, S1 and KH subunits [20].

Access to the PNPase active sites is through the central channel, which can accommodate single-stranded RNA with some structural adjustment of a constricted aperture at the channel entrance. The S1 and KH RNA-binding domains are located on the surface of the trimer near the aperture, but they are highly dynamic so that their detailed structures have been difficult to resolve. Recently, the KH domains were defined in the crystal structure of human PNPase [19], but only partial backbone traces of the KH and S1 domains could be modelled in the *S. antibioticus* structure [17], guided by the structures of the isolated *E. coli* PNPase S1 domain [21] and the human vigilin KH domain. None of the peptide backbone for the KH or S1 domains could be traced for the *E. coli* structure [22].

Structural studies have explained many of the enzymatic properties of PNPase. First, the enzyme cleaves single-stranded RNA in the 3′–5′ direction with ‘processive’ behaviour, meaning that once the first cleavage is initiated, cutting continues along the same substrate until it becomes stalled by RNA secondary structure [17]. Processivity is accounted for by the ring-like quaternary organization of the trimer, which presumably retains the substrate once engaged. The three catalytic sites of the PNPase trimer reside in shallow grooves within the central channel, where a bound metal is proposed to assist inorganic phosphate attack on the 3′ phosphodiester bond of single-stranded RNA, releasing a single nucleoside diphosphate with each successive cleavage. The crystal structure of *E. coli* PNPase with manganese ion bound corroborates the key role of conserved acidic residues in the catalytic mechanism [23].

Structural data also reveal that the constricting aperture at the entrance to the central channel is formed by a loop containing highly conserved phenylalanines that make aromatic stacking interactions with the bases of engaged RNA [23]. The proximity of the S1 and KH domains to the entrance suggests that the RNA-binding domains may assist substrate delivery through the aperture and into the central channel, which is consistent with catalytic behaviour of truncated PNPase that lacks these domains [24]. However, it is not known how the KH and S1 domains engage and guide the substrate through the narrow entrance. It is also not known how RNA is orientated directionally, such that the 3′ end enters first, and how the substrate is threaded along to the active site. Our crystal structures of RNA-bound and apo bacterial PNPase address these questions and suggest that a mechanical ratcheting-like movement may help to channel the substrate to and from the active sites.

The PNPase studied here is from the bacterium *Caulobacter crescentus*, which has been established as a model system to investigate cell cycle progression, because populations can be readily prepared that are synchronized in the cell cycle [25]. Recently, *C. crescentus* PNPase was shown to form part of a multi-enzyme RNA degradosome assembly, to which PNPase is recruited through interaction with a short conserved motif at the C-terminus of the endoribonuclease RNase E [26]. The crystal structure presented here of *C. crescentus* PNPase in complex with the conserved peptide motif explains how PNPase interacts with RNase E to form an assembly involved in post-transcriptional regulation, and mutational analysis *in vitro* and *in vivo* corroborates the importance of two conserved tryptophan residues at the C-terminus of RNase E for this interaction.

## 3. Material and methods

### 3.1. Cloning, expression and purification of *Caulobacter crescentus* PNPase and GST–GWW peptides

Genomic DNA was isolated from *C. crescentus* PNPase strain NA1000 using a GenElute bacterial genomic DNA kit (Sigma). The *pnp* gene (CC0034) was amplified from the isolated genomic DNA using the forward primers: ccPNP_pGEX6p1.fw or ccPNP_6xHIS.fw, and the reverse primer: ccPNP.rev (primers summarized in table 1). Complementary forward and reverse primers corresponding to the 3′ end of the *rne* gene, encoding the PNPase recognition peptide (TAPPEKPRRGWWRR) and alanine mutations of the tryptophan codons were synthesized and annealed.

**Table 1.** Primers used in this study. Nucleotides in bold represent mutated codons.

primer	sequence (5′−3′)
ccPNP_pGEX6p1.fw	GCGGATCCATGTTCGATATCAAACGCAAGACG
ccPNP_6xHIS.fw	GCGGATCCGATGTTCGATATCAAACGCAAGACG
ccPNP.rev	CGGCGGCCGCTTACGCCTCTTCGGCCGCCGCTTCC
GWW_pGEX.fw	CCGGATCCACCGCGCCGCCCGAAAAGCCCCGTCGGGGCTGGTGGCGCCGGTAACTCGAGCG
GWW_pGEX.rev	CGCTCGAGTTACCGGCGCCACCAGCCCCGACGGGGCTTTTCGGGCGGCGCGGTGGATCCGG
GAW_pGEX.fw	CCGGATCCACCGCGCCGCCCGAAAAGCCCCGTCGGGGC**GCG**TGGCGCCGGTAACTCGAGCG
GAW_pGEX.rev	CGCTCGAGTTACCGGCGCCA**CGC**GCCCCGACGGGGCTTTTCGGGCGGCGCGGTGGATCCGG
GWA_pGEX.fw	CCGGATCCACCGCGCCGCCCGAAAAGCCCCGTCGGGGCTGG**GCG**CGCCGGTAACTCGAGCG
GWA_pGEX.rev	CGCTCGAGTTACCGGCG**CGC**CCAGCCCCGACGGGGCTTTTCGGGCGGCGCGGTGGATCCGG
GAA_pGEX.fw	CCGGATCCACCGCGCCGCCCGAAAAGCCCCGTCGGGGC**GCGGCG**CGCCGGTAACTCGAGCG
GAA_pGEX.rev	CGCTCGAGTTACCGGCG**CGCCGC**GCCCCGACGGGGCTTTTCGGGCGGCGCGGTGGATCCGG
3713_SpeI-700upcc1877-fw	AGTTACTAGTCATTGGGCAGCGCGATGAC
3714_Upcc1877-3xflag-rv	CACCGTCATGGTCTTTGTAGTCCATAAAGGAAGTCTCCGCGGCG
3715_Upcc1877-3xflag-fw	CGCCGCGGAGACTTCCTTTATGGACTACAAAGACCATGACGGTG
3746_3xflag-cc1877-rv	CGTCGATCAGCATCTTCTTCGACATTTTATCGTCGTCATCTTTGTAGTCG
3716_3xflag-cc1877-fw	CGACTACAAAGATGACGACGATAAAATGTCGAAGAAGATGCTGATCGACG
3718_700cc1877-NheI-rv	TCAAGCTAGCGGTACTCATAGTCGCGCTTG
3719_SpeI-700cc1877-fw	AGTTACTAGTGATGATGAAGGCGGTCGTCG
3736_2664cc1877-stop-down-rv	CGGGCCGTTGTCATTCGCCCTTAGGGCGGCGCGGTGATCTCGTTCG
3737_2664cc1877-stop-down-fw	CGAACGAGATCACCGCGCCGCCCTAAGGGCGAATGACAACGGCCCG
3724_700downcc1877-NheI-rv	TCAAGCTAGCCCGGACTGGCCGGTGGCTTCCAGGAAGCC

The *pnp* gene was ligated into pGEX-6p-1 (GE Healthcare) and pET-DUET (Merck Biosciences) expression vectors to generate N-terminal GST and 6xHis-tagged versions of PNPase, respectively, *rne* gene fragments encoding GWW peptides were ligated into pGEX-6p-1. Transformed *E. coli* cells were grown in 2x YT media at 37°C until OD 600 nm reached 0.4, and then induced with 1 mM isopropyl beta-d-1-thiogalactopyranoside (IPTG) at 18°C for 15 h (PNPase) or 37°C for 3 h (GST–GWW peptides). Cells were harvested and resuspended in either pGEX-lysis buffer (20 mM Tris–HCl pH 7.5, 200 mM NaCl, 1 mM dithiothreitol) supplemented with 5 mM EDTA, or 6xHis-lysis buffer (20 mM Tris–HCl pH 7.5, 500 mM NaCl, 5 mM imidazole) supplemented with complete EDTA-free protease inhibitor (Roche). Resuspended cells were lysed with an Emulsiflex cell disruptor (Aviston) and clarified by centrifugation at 30 000*g* for 30 min at 4°C. For GST–PNPase and GST–GWW peptides, the lysate was then loaded onto a glutathione sepharose column, washed with lysis buffer and eluted with lysis buffer supplemented with 50 mM reduced glutathione. GST–GWW peptides were stored at −80°C. GST–PNPase was incubated with Precission Protease (GE Healthcare) at 4°C for 15 h, concentrated and loaded onto a Sephacryl 200 (GE Healthcare) and eluted in GST-lysis buffer. For 6xHis–PNPase, the lysate was loaded onto a 5 ml HisTrap HP column (GE Healthcare), washed with 6xHis-lysis buffer, then eluted in 6xHis-lysis buffer supplemented with 500 mM imidazole. Fractions enriched in PNPase were supplemented with 50 mM sodium phosphate at 37°C for 1 h to drive phosphorolysis of the co-purifying RNA, and then reapplied to Sephacryl 200. The RNA content of the purified sample at this stage was estimated to be 15 per cent by UV spectroscopy (260 : 280 nm ratio of 1.55). This is consistent with profiles from analytical ultracentrifugation, suggesting a mass excess above the expected trimer corresponding to approximately 20 nucleotides. Avidly bound RNA was removed by heparin column equilibrated with loading buffer (20 mM Tris–HCl pH 7.5) and developed with a linear gradient of elution buffer (20 mM Tris–HCl pH 7.5, 1 M NaCl).

### 3.2. Electrophoretic mobility shift assay

#### 3.2.1. Polynucleotide phosphorylase

GST–GWW binding experiments were performed in a buffer composed of 20 mM Tris–HCl, pH 7.5 and 200 mM NaCl. An approximate fourfold molar excess of GST–GWW, GST–GWA, GST–GAW or GST–GAA was mixed with PNPase and incubated at room temperature for 10 min prior to analysis by 8 per cent acrylamide non-denaturing polyacrylamide gel electrophoresis (PAGE).

### 3.3. Crystallization and data collection

RNA-free and RNA-bound PNPase were concentrated to approximately 15 mg ml^−1^. The *C. crescentus* PNPase recognition peptide from RNase E (EKPRRGWWRR; Cambridge Research Biochemicals) was mixed at a 2 : 1 molar excess with apo-PNPase immediately prior to crystallization. The protein–peptide and protein–RNA complexes were mixed with an equal volume of crystallization buffers. Crystals were grown by sitting droplet vapour diffusion at 18°C and appeared after approximately one week for all crystal forms. Crystals for RNA-bound PNPase appeared in 24 per cent w/v PEG 3350, 0.1 M bis–Tris (pH 5.5), 0.1 M ammonium acetate. Hexagonal crystals of apo-PNPase appeared in 17 per cent w/v PEG 3000, 0.1 M trisodium citrate pH 5.5. Rhombohedral crystals of apo-PNPase appeared in 19 per cent w/v PEG 3350, 0.15 M dl-malic acid. Crystals were harvested in reservoir solutions supplemented with 25 per cent v/v glycerol as cryoprotectant, then flash frozen in liquid nitrogen. Diffraction data were collected at 100 K using stations IO2 and I24 at Diamond Light Source, Didcot, UK. The crystal used for the RNA-bound dataset was treated by rapid temperature annealing. Data were processed with MOSFLM [27] and merged with SCALA [28].

### 3.4. Modelling and refinement

All PNPase structures were solved by molecular replacement with the program Phaser [29] using a single protomer of the RNase PH-like core of the *E. coli* PNPase as an initial search model (PDB code 3GME [23]). For the hexagonal apo-form, this generated a trimer via crystal lattice symmetry, whereas for the RNA-bound structure, a trimer was completed by fitting two additional PNPase protomers into the crystal asymmetric unit. In both cases, the electron density maps calculated from the initial models revealed clear density for the KH domains. Of the available KH domain structures, the human vigillin protein (PDB 1VIH) has the greatest predicted structural homology to the *C. crescentus* PNPase KH domain using the FUGUE server (http://tardis.nibio.go.jp/fugue) [30]. The human vigillin KH domain was used as a guide to manually build the *C. crescentus* KH domain in the clearest density of the early electron density maps. The models were improved iteratively with refinement using REFMAC [31] and BUSTER [32], followed by manual rebuilding using COOT [33]. For the RNA-bound structure, the improved KH model was placed into the density at the two other corresponding positions. Non-crystallographic symmetry restraints were used for the RNase PH-like core, the KH domain and the helical domain. The density for the RNA became more clearly defined during the iterative building procedure, and was included in the model at full occupancy where it contacts the KH domain, or at partial occupancy where it stacks on F77 at three conserved FF loops at the aperture of the central channel (see electronic supplementary material, figures S1 and S3). The RNA was removed from the model, which was then refined with REFMAC [31], and then used to guide rebuilding. The final model includes water molecules and phosphate ions at the active sites. For the hexagonal apo-form of PNPase, electron density could also be clearly seen for the S1 domains. The nuclear magnetic resonance spectroscopy (NMR) model of isolated *E. coli* PNPase S1 domain [21] was used as a guide to manually build the S1 domain into this density. For the rhombohedral and hexagonal crystal forms of apo-PNPase, additional electron density could also be seen corresponding to the GWW peptide. Nine residues of the peptide were built manually into this density for the rhombohedral crystal form, and then used to guide the modelling of the peptide in the hexagonal crystal form. The models have been deposited with the protein structural database with entry codes 4AM3, 4AID and 4AIM.

### 3.5. Construction of *Caulobacter crescentus* strains

DNA sequences upstream of cc1877 (*rne*), a triple-flag sequence, and the beginning of the *rne* gene were PCR amplified using the primer pairs 3713_SpeI-700upcc1877-fw and 3714_Upcc1877-3xflag-rv, 3715_Upcc1877-3xflag-fw and 3746_3xflag-cc1877-rv, or 3716_3xflag-cc1877-fw and 3718_700cc1877-NheI-rv, respectively. The three fragments were connected via overlap extension PCR, and the product was digested using *Spe*I and *Nhe*I restriction enzymes. Ligation of this insert with pNPTS138, digested with the same restriction enzymes, resulted in pIH79. For the construction of pIH84, the DNA flanking the sequence encoding the PNPase-binding site of RNaseE (last 10 amino acids) were amplified using the primer pairs 3719_SpeI-700cc1877-fw and 3736_2664cc1877-stop-down-rv, or 3737_2664cc1877-stop-down-fw and 3724_700downcc1877-NheI-rv, respectively. The fragments were connected by overlap extension PCR, the product was cut with *Spe*I and *Nhe*I restriction enzymes, and ligated with the pNPTS138 vector.

The *C. crescentus* chromosomal *rne* gene was replaced by versions encoding an N-terminal flag tag or lacking in addition the PNPase-binding site. The suicide plasmids pIH79 and pIH84, respectively, were introduced into wild-type *C. crescentus* cells by conjugation, single recombinants were selected by growth on kanamycin and double recombinants by growth on sucrose plates.

## 4. Results

### 4.1. Structural organization of a complete polynucleotide phosphorylase

*Caulobacter crescentus* PNPase was co-crystallized with a short peptide corresponding to its recognition motif from RNase E [26] in two different space groups (P6_3_ and R3; table 2). The structures were solved by molecular replacement using the conserved RNase PH-like core of the *E. coli* enzyme. The asymmetric unit of the rhombohedral crystals contains three PNPase protomers, while the hexagonal crystal form contains a single protomer. Although no electron density was apparent for the KH and S1 domains of PNPase in the rhombohedral crystal form, clear density was present for these domains in the hexagonal crystal. The KH and S1 domains were manually built into this density using as guides the NMR model of the isolated PNPase S1 domain and the X-ray structure of a KH domain from human vigillin.

**Table 2.** Crystallographic data collection and refinement.

	RNA-bound (4AM3)	Apo + GWW peptide (4AID)	Apo + GWW peptide (4AIM)
space group	P22_1_2_1_	R3 (H3 setting)	P6_3_
unit cell dimensions (Å)	*a* = 93.64, *b* = 112.06, *c* = 236.22	*a* = 157.44, *b* = 157.44, *c* = 302.38	*a* = 97.33, *b* = 97.33, *c* = 191.91
crystallization conditions	24% w/v PEG 3350, 0.1 M bis–Tris pH 5.5, 0.1 M ammonium acetate	19% w/v PEG 3350, 0.15 M dl-malic acid	17% w/v PEG 3000, 0.1 M trisodium citrate
resolution (Å)	35.0–3.00 (3.16–3.00)	30.0–2.60	40.0–3.30 (3.48–3.30)
light source, wavelength (Å)	diamond IO2, 0.9795	diamond IO2, 0.9795	diamond I24, 0.9778
unique reflections	50 090	41 196	15 391
multiplicity	4.5 (4.5)	6.3 (6.2)	4.7 (4.6)
completeness (%)	99.2 (99.6)	93.2 (94.8)	99.3 (99.2)
intensity/*σ*	9.0/2.7	5.5/2.4	7.8/2.4
*R*_merge_ (%)	12.9 (52.3)	21.6 (57.6)	13.6 (65.0)
Wilson B factor (Å^2^)	66.1	52.1	86.6
refinement parameters			
resolution (Å)	35.0–3.00	30.0–2.60	40.00–3.30
R-factor	0.210	0.209	0.186
R-free	0.254	0.255	0.260
number of reflections used	47 529	85 978	15 528
total number of atoms	14 015	13 030	4957
total number of amino acid residues	1864	1708	706
total number of water, phosphate	54, 6	207, 3	2, 1
total number of RNA bases	12	0	0

The structure of the complete PNPase reveals how the KH and S1 RNA-binding domains are spatially arranged in relation to the RNase PH-like catalytic core (figure 1*b*). The three KH domains are positioned over the entrance to the catalytic core like an umbrella, with the RNA-binding GSGG loops (residues 572–575) forming a secondary entrance aperture. We will return again to discussions of this loop in §4.3. The S1 domains adopt a splayed conformation, and the conformation is constrained by the crystal packing (see electronic supplementary material, figure S6). We suggest that the S1 domains are flexibly tethered to the KH domains, and that they potentially could manoeuvre to capture RNA substrates as part of a molecular fly-casting mechanism. The RNA-binding surfaces of the S1 domains face the molecular threefold axis at the centre of the PNPase trimer, and it is envisaged that S1 domains could clamp down in concert on RNA targets.

### 4.2. An RNA-bound state for polynucleotide phosphorylase

**Figure 2.**
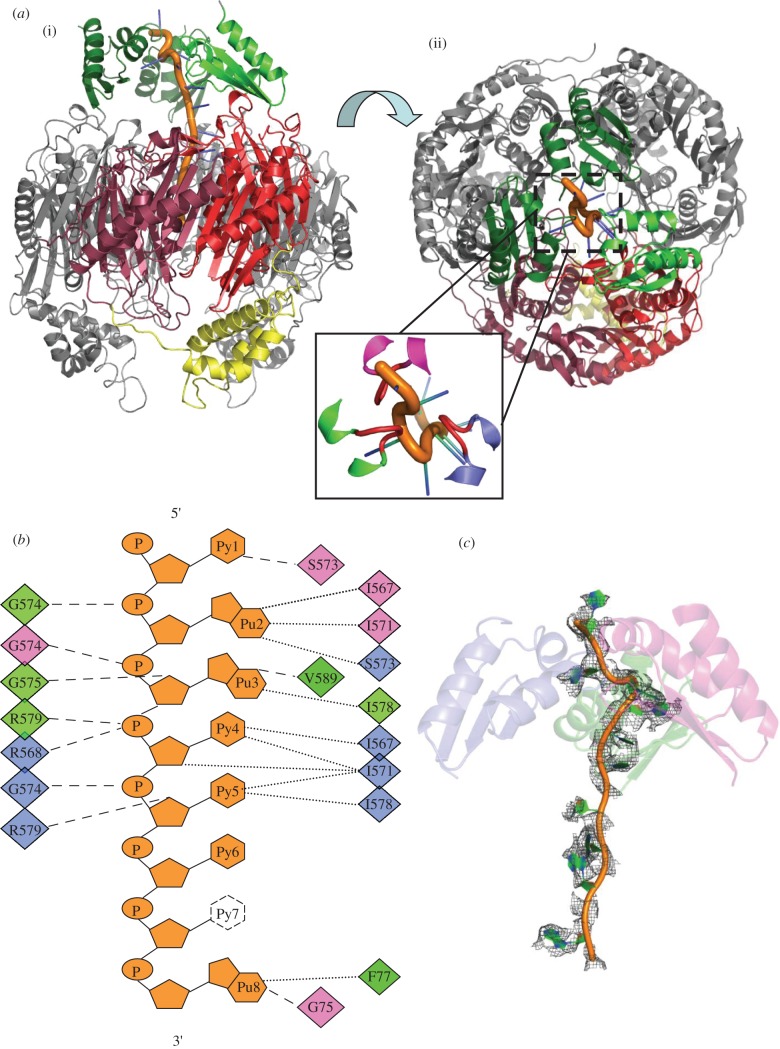
Interactions of *Caulobacter crescentus* PNPase with RNA. (*a*) (i) and (ii) correspond to the perspectives shown in figure 1*b*, with the three KH domains coloured green. The inset is an expanded view with the GSGG loops coloured red. (*b*) Schematic of the contacts with the RNA. Residues involved in phosphate backbone hydrogen bonding are shown on the left. Residues forming van der Waals contacts are in the far right margin, and the residues hydrogen bonding to bases or sugar 2′-OH groups are in the middle-right group. The diamonds are colour coded for the three PNPase chains. All contacts shown are from the KH domain or from the FFKR loop at the entry aperture of the central channel. Base 7 is disordered and has not been modelled in the final structure. (*c*) Electron density map for endogenous RNA chain. An omit map of the RNA chain contoured at 1*σ* is shown as grey mesh. RNA is shown as orange and green cartoon, and the three KH domains are shown as semi-transparent cartoons, coloured separately as in figure 2*b*.

In the course of preparing recombinant *C. crescentus* PNPase, it was found that oligonucleotide originating from the *E. coli* expression host remained avidly associated with the protein during chromatographic purification. Extensive incubation with phosphate to promote phosphorolysis, followed by gel filtration, liberated nucleoside diphosphate; however, the limit digest product still remained associated with the PNPase. On the basis of absorbance at 260 nm, an estimated 20–30 nucleotides were bound to each PNPase trimer. It was possible to liberate a fraction of the protein from RNA by chromatography, and this material was used to prepare the apo-form crystals described in §4.1. Crystals of the RNA-bound material were obtained in space group P22_1_2_1_ that contain a PNPase trimer in the asymmetric unit (table 2). The structure was solved by molecular replacement using as search models the RNase PH-like core and a KH domain from the apo structure. The other two KH domains were found in the unbiased maps. It was also clear from the early electron density maps that RNA was present and interacting with the KH domains (figure 2*a*). The KH domain is structurally similar to the human poly(C) binding protein, and the GxxG loop makes similar contacts with nucleic acids (electronic supplementary material, figure S2).

Although purines could be distinguished from pyrimidines in the modelled RNA, it was not possible to definitively identify the base sequence. The refined crystal structure revealed 12 RNA nucleotides, and while it was anticipated that 20–30 oligonucleotides would be present in the structure based on spectroscopic absorbance, the RNA may have been partially degraded during crystallization or may be extensively disordered in some places. The S1 domains were not visible in this structure, although the presence of these domains in the crystals was confirmed by SDS–PAGE of harvested crystals. The S1 domains are therefore apparently disordered in the RNA-bound structure.

To evaluate the interactions with a defined RNA species, crystals of RNA-bound PNPase were obtained by co-crystallization of apo-PNPase with a short synthetic RNA (27mer 5′-GUUUAUUGCCGUUCUUGUUAUAUGCCUU), corresponding to a portion of *E. coli* MicC small non-coding RNA. The protein:synthetic RNA complex crystallized in the same unit cell and space group as the endogenous RNA complex crystals. The structure with the synthetic RNA corroborates the interactions seen with the endogenous RNA; however, the resolution and map quality were marginally better for data collected from crystals of PNPase in complex with endogenous RNA, and the following analysis is based on data from the endogenous RNA co-crystal.

### 4.3. K-homology domain and aperture interactions with the RNA

The three KH domains (residues 556–619) interact with the RNA in the manner of three overlapping hands grasping a length of rope. These contact sites will be described as upper, middle and lower positions according to the distance from the RNase PH-like core (figure 2*a*). A key set of interactions is made with the phosphate backbone of RNA by a glycine-rich loop (GSGG, residues 572–575) separating two α-helices, as shown in figure 2*a* and summarized schematically in figure 2*b*. At the most distal contact with the RNA, S573 of the middle GSGG loop forms a hydrogen bond to the 2′-OH of the first base, whereas the amide of G574 (upper) interacts with phosphate of the second nucleotide. The base of the second nucleotide interacts with S573 of the lower KH domain and, in turn, G574 of the middle domain contacts the phosphate of the third base. Finally, the fourth and fifth bases are buried against an exposed hydrophobic patch of the lower KH domain, whereas G574 contacts the phosphate of the fifth base. The RNA follows a spiralling trajectory, with bases on the outside and phosphate backbone innermost. Portions of the density indicate disorder in the backbone, and this is also apparent in an omit electron density map of the entire RNA chain, which corroborates the key contact points with the protein (figure 2*c*).

The path of the RNA continues from the KH ensemble to the aperture of the central channel, where a base stacks on the aromatic side chain of the conserved FF loop (electronic supplementary material, figure S3). Here, partial density for stacked bases is seen at each FF loop, and probably results from averaging of three separate conformations of the RNA, rather than having three separate chains, because the opening is not sufficiently large to accommodate more than one polynucleotide chain. The overlay of the FF loops of *C. crescentus* (grey) and *E. coli* (black; PDB 3GCM) with bound RNA shows that the interaction is similar in both enzymes. Some density could be seen at a protomer interface adjacent to the FF loop and may be a binding site for one nucleotide base. Some residual density could be seen at all three active sites, but it was not of sufficient quality to enable modelling. The pathway of the RNA continuing from the narrow FF-loop aperture to the active site is not resolved in the electron density maps, but broken density indicates that a disordered polymer might bridge those two points.

### 4.4. Determinants of RNA directionality

The question naturally arises how the RNA is oriented so that the 3′ end is directed towards the PNPase aperture. We do not observe any contacts with the 3′ end of the RNA that might contribute to directionality of the substrate, and because our structure represents effectively a snapshot of a late stage of substrate entrapment, we cannot comment on whether the 3′ end is explicitly recognized during the early capture stage. However, the structure suggests that the 3′–5′ polarity of the substrate originates from the interactions of the GSGG loops with the RNA sugar–phosphate backbone by the KH domains (figure 2*b*). These interactions are reinforced cooperatively through the spatial organization of the KH domains, mediated by favourable inter-domain contacts.

### 4.5. Potential rotary movement of the K-homology domains as a group

**Figure 3.**
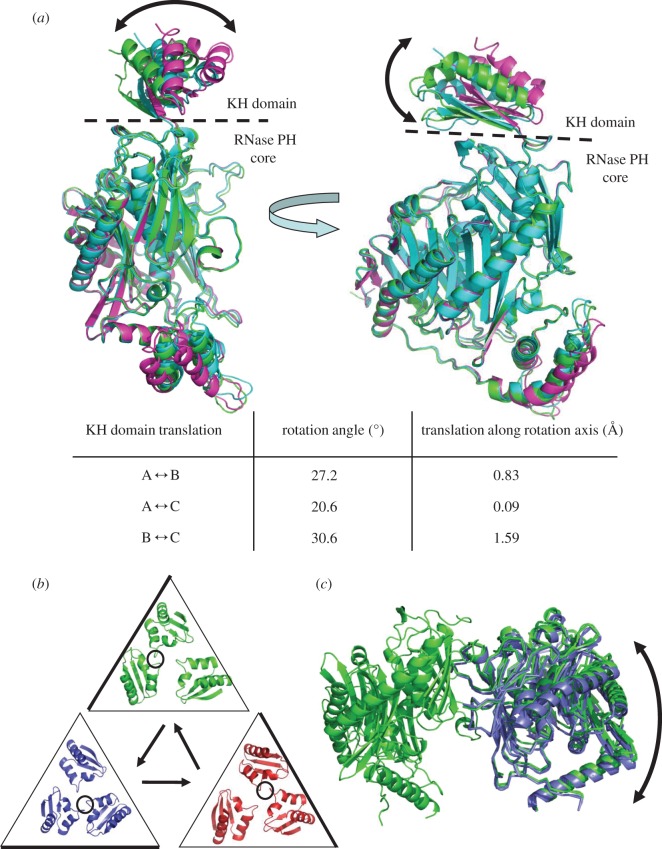
Movement of the K-homology (KH) domain ensemble and non-equivalence in the polynucleotide phosphorylase ring. (*a*) The location of the KH domain varies with respect to the RNase PH core, which is the reference frame for the overlay. The boundary between KH domain and RNase PH core is shown as a dashed line. (*b*) Rotational displacement of the group of KH domains on the surface of the PNPase core. The circularly permuted locations of the KH domains are coloured blue, red and green, and shown inside triangles. The GxxG loops making the majority of the contacts with the RNA are circled and the side of the triangle nearest to the GxxG loops is shown as bold. Whether there is a direction of rotation associated with polymerization or degradation is not known. Accompanying movies illustrate these structural non-equivalences (see electronic supplementary material). (*c*) Quaternary structural change in the RNase PH-like core. The trimeric core has been circularly permuted. Two protomers on the reference subunit are shown in green, and protomer movement with respect to the reference is shown in blue. The KH and all-helical domains have been removed for clarity.

The KH domains adopt non-equivalent orientations with respect to the RNase PH-like core, and each domain contacts the RNA at different points (figure 2*a*). The ‘lower’ KH domain is packed against the proximal face of the RNase PH core, with interactions between D590 (KH domain) and G361 (RNase PH core), as well as an interaction between S600 (KH domain) and T360 (RNase PH core). Non-equivalent interactions between the KH domains and catalytic core are seen in the KH domains at the ‘middle’ and ‘upper’ positions, namely the I557/P555 (KH domain) and Y425 (RNase PH core) contact. In the reference frame of the RNase PH trimer, the ensemble of KH domains is displaced from the central threefold axis, and defines three different states (figure 3*a*). Analysis of the displacement of the KH domains by DynDom (http://fizz.cmp.uea.ac.uk/dyndom/) [34] reveals that residues 555–557 act as a mechanical hinge, allowing large rotational movement of the KH domains (summarized in table inset in figure 3*a*; the rotation axes are represented in the electronic supplementary material, figure S4). Assuming all three KH domains can alternate between the three different orientations, circularly permuting through these states gives the impression of rotary displacement of the KH ensemble (shown in a molecular graphics movie available in electronic supplementary material).

The hypothesis that the KH domains can undergo marked movement is supported by the recent X-ray crystal structure of human PNPase [19]. In the human PNPase structure, the KH domains are resolved, but comparison of this structure with the *C. crescentus* structures reveals that they adopt strikingly different conformation with respect to the catalytic core (electronic supplementary material, figure S5*a*). The RNA-binding GxxG loops are spatially distant in the human structure, presenting an apparent ‘open’ conformation for the KH aperture (electronic supplementary material, figure S5*b*). Perhaps such presentation may aid substrate capture.

### 4.6. Quaternary change in the RNase PH-like core

Structural non-equivalence is also observed in the RNase PH-like core, which can be seen most clearly by circularly permuting the subunits in a common reference frame of one of the subunits (figure 3*c*; electronic supplementary material, movie S2). With respect to the fixed subunits in the reference frame of the RNase PH fold, the centre of mass of the neighbouring subunits is displaced by roughly 2 Å. Inspection of the interfaces between the protomers suggests that there is a continuum of small local adjustments involved and not a simple binary switch, as found, for example, in the quaternary structural transition of haemoglobin [35]. The movement through the three states may be associated with changes in the size of the constricting aperture made by the FF loops, and linked also with the opening and closing of a small interfacial pocket adjacent to the aperture that may fit a base. Such movements in the protein, associated with small quaternary changes, may be coupled with the displacement of the RNA through the aperture.

### 4.7. Interaction of polynucleotide phosphorylase with the RNase E recognition peptide

**Figure 4.**
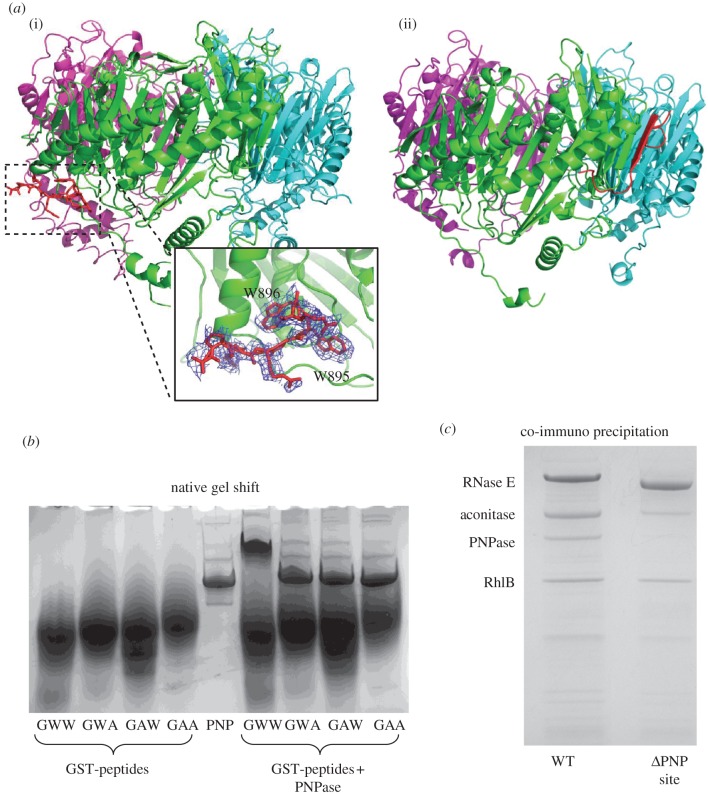
Recognition of RNase E by *Caulobacter crescentus* PNPase. (*a*) (i) Interaction of *C. crescentus* PNPase with RNase E recognition peptide. (ii) Interaction of *E. coli* PNPase with the recognition site in RNase E (pdb entry 3GME). The GWW motif, conserved in the *α*-proteobacterial RNase E, is bound at the opposite face of the PNPase ring from the surface exposing the SI and KH domains (at the top, not shown). The GWW motif peptide is shown in red. The inset is an expanded view showing the GWW peptide and an omit map for this peptide. (*b*) Mutation of the GWW motif *in vitro*. GST fusions of the PNPase recognition peptide were expressed with alanine mutations of the conserved tryptophan residues, and the ability to bind to PNPase was assessed by an electrophoretic mobility shift assay. (*c*) Deletion of the PNPase-binding site from RNase E *in vivo*. The PNPase recognition peptide was deleted from chromosomal RNase E, and the resulting pull-down of degradosome components was assessed by SDS–PAGE.

It has previously been shown that *C. crescentus* PNPase interacts with the endoribonuclease RNase E to form part of an RNA–degradosome complex [26]. This interaction is mediated by a short segment at the C-terminus of RNase E containing a GWW motif that is strikingly conserved in RNase E of the α-proteobacteria. To examine further the importance of the conserved GWW motif in the PNPase recognition peptide, we substituted the tryptophan residues individually and in combination with alanine, and assessed the impact on PNPase binding using a native electrophoretic mobility shift assay (figure 4*b*). GST fused to the PNPase recognition peptide (TAPPEKPRRG*WW*RR) was mixed at an approximate fourfold molar excess with PNPase, and the mixture was then resolved on a native gel. The band corresponding to free PNPase was completely shifted to a slower migrating species in the presence of the wild-type GST–GWW peptide. However, when either tryptophan was mutated to alanine the formation of the complex with PNPase was severely disrupted.

The impact of the mutations of RNase E:PNPase interactions was evaluated *in vivo*. For these experiments, a strain of *C. crescentus* was generated with a flag tag fused to the N-terminus of genomically encoded RNase E and a deletion of the putative PNPase recognition GWW motif (figure 4*c*). When the GWW motif is absent from RNase E, the amount of PNPase retrieved from the pull-down experiment is greatly reduced, if not totally absent, whereas the other degradosome components (RNase E, aconitase, DEAD-box helicase RhlB) are present at levels similar to pull-downs from the wild-type strain.

Crystals of RNA-free PNPase with recognition peptide (KPRRGWWR) revealed clear density for the peptide in both the hexagonal and rhombohedral crystal forms of PNPase. For the rhombohedral crystal form, eight residues of the peptide could be modelled into this density. The GWW motif occupies a hydrophobic pocket formed by the loop connecting helix α2 and strand β7, and the linker region between helices α5 and α6 on the surface of PNPase (figure 4*a*(i)). In the hexagonal crystal form, the GWW peptide additionally forms crystal packing contacts with the S1 domain of a neighbouring molecule (electronic supplementary material, figure S6). For the *E. coli* homologues, the interaction involves the RNase E peptide becoming an additional strand with the exposed edge of a sheet from PNPase [23]. This recognition site on the *E. coli* PNPase is spatially distant from the corresponding location of the GWW peptide recognition site on the surface of *C. crescentus* PNPase (figure 4*a*(ii)).

## 5. Discussion

The crystal structures of *C. crescentus* PNPase reveal for the first time how the RNA-binding KH and S1 domains are spatially organized in relation to the catalytic core of the enzyme, and how single-stranded RNA is engaged by the KH domains on the surface of the RNase PH-like core. A functionally important signature of KH domains is the GxxG motif, which is the site of interaction with a tetranucleotide for many proteins harbouring KH domains [36]. In PNPase, the motif is also engaged to RNA, but contacts fewer bases and interacts mostly with the phosphosugar backbone. RNA interactions are made by the serine and glycine residues sandwiched between the conserved glycines, and additionally by some flanking residues. Mutation of the first glycine of the GxxG loop to aspartate in *E. coli* PNPase decreases affinity for RNA [37,38]. The crystal structure presented here rationalizes this observation, suggesting that the aspartate sterically hinders RNA binding by the GxxG loop and is charge-repulsive for the phosphate backbone.

The KH domains are flexible in the bacterial PNPase structures and can cooperate through RNA interactions consolidated by a few weak salt bridge contacts. Notably, there is a salt bridge between the conserved residues D593 (‘upper’ domain) and R579 (‘middle’ domain; annotated in figure 1*b*), which is absent from the other two KH domain interfaces. The path of the RNA can be traced from the KH domains to the FF loop at the entrance to the central processing core of PNPase; however, there is no clear density to connect the active site and the RNA at the aperture formed by the FF loops.

**Figure 5.**
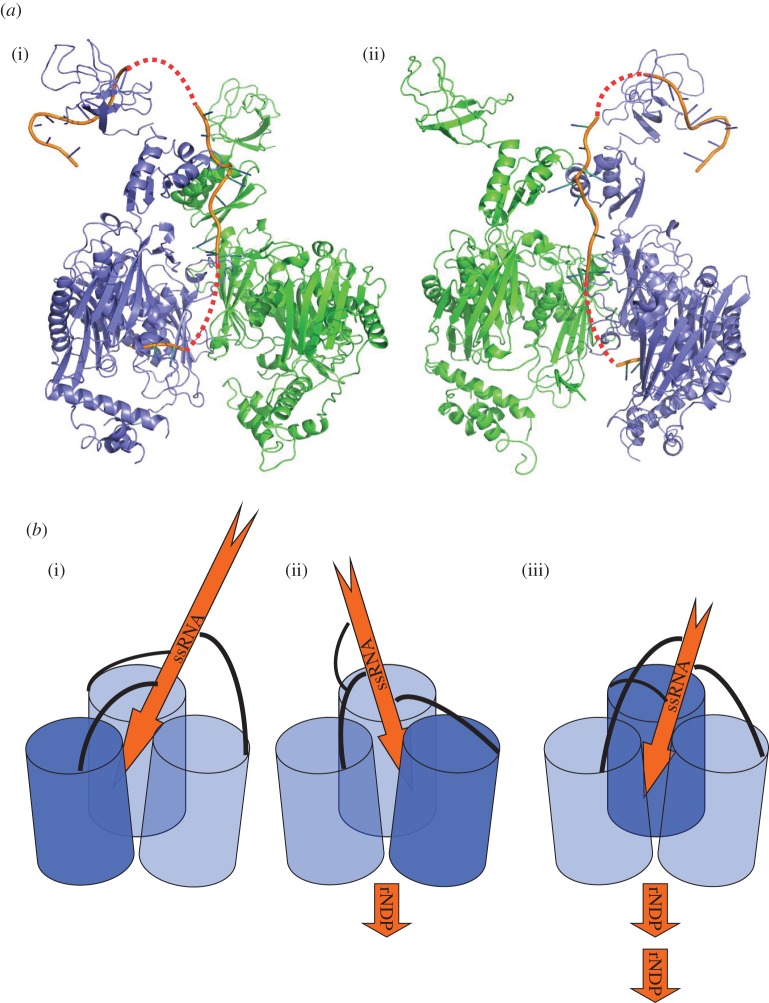
The path of the RNA in polynucleotide phosphorylase (PNPase). (*a*) A speculative model for the path of the RNA in the bacterial PNPase from the S1 domains to the active site. (i,ii) Two views rotated approximately 120°; for clarity, only two PNPase protomers are shown (green and blue). RNA bound to *C. crescentus* PNPase is shown as orange cartoon. RNA modelled at the active site is based on the position of RNA bound to the structurally homologous archaeal exosome (3M7N). RNA bound to the S1 domains is based on the position of RNA bound to the S1 domain of *E. coli* RNase E (2C0B). Predicted links between the RNA segments are shown as a dashed red line. (*b*) Schematic of the proposed threading mechanism. PNPase core protomers are depicted as blue cylinders, KH domains as black curved lines and single-stranded RNA as orange arrows. (i,ii,iii) The proposed rotary movement of the KH domains threading the RNA substrate to active sites of adjacent protomers, with the dark blue protomer representing the active site currently engaging the substrate. The model is speculative and proposes that the RNA may be bound and cleaved in three different active sites in the PNPase trimer.

We have prepared a speculative model for the path of single-stranded RNA by combining the crystal structures of the *C. crescentus* PNPase and of an archaeal exosome with a short segment of RNA leading from the active site to the proximal aperture (figure 5*b*). In the RNA-free structure of PNPase, the S1 domains were resolved but were poorly ordered and could not be resolved in the RNA-bound structure, and as such their role in substrate recognition and processing remains to be elucidated. In the model shown in figure 5, we have used the RNA bound to the S1 domain of *E. coli* RNase E as a guide to speculate on the path of the nucleic acid from one extreme end of the PNPase trimer to the active site.

One salient feature of the structure is the asymmetry of the RNA-bound PNPase. We suggest that the asymmetry in PNPase is functionally important, and enables substrate channelling and communication between the active sites. We envisage that substrate is channelled by a coupling mechanism, which would link RNA binding by the RNA-binding domains with displacement of the polynucleotide through a constricting aperture to thread to the active sites (figure 5*b*). The up–down movement of RNA substrates through the central channel could be favoured by binding and displacement of the bases at the FF-loops (figure 2*c*) and at a small pocket adjacent to the aperture inside the channel. The threading mechanism is thus envisaged to be a biased ratchet, in which the RNA moves along because of the slightly favoured binding energy of the 3′ terminus for the active site [18], or a strongly coupled mechanism, in which the quaternary structural changes actively drive the displacement.

Ratcheting of the substrate would require the bottom KH domain to disengage from the RNA chain following each phosphorolytic event and re-engage at the upper position for the process to continue. The relatively weak interactions between the KH domains and the RNA may allow for such rapid association and dissociation. Given the knobbly nature of the contacting surfaces, it seems that the entire chain must be displaced from the surfaces before it could move along. In this ratcheting-like movement, the end of the RNA could visit one of the neighbouring catalytic sites. Whether the mechanism involves obligate movement between active sites with a defined rotational sense or random movement between sites remains to be investigated. However, communication between active sites of the RNase PH-like core could orchestrate their activities, so that product from one active site shuttles to one of the adjacent free sites. The mechanical displacement of the strand may be linked with substrate binding at one of the vacant active sites, which would be either inorganic phosphate in the case of the degradative direction, or NDPs in the case of the reverse (random polymerization) direction. Accordingly, the energy for the displacement would originate from the free energy of binding of phosphate or NDP at one of the vacant active sites.

The model for mechanical coupling in PNPase might explain the puzzling observation that PNPase cleaves RNA at similar rates regardless of length over a certain threshold size of six to nine nucleotides, and that cutting of the longer substrates is inhibited by short RNA (such as 6-mers) [39,40]. This inhibition can arise if the short RNAs gain access to the active sites and occlude the movement of substrate between successive sites. If a similar effect occurs in the exosome, then it would explain why long substrates are cleaved in a processive manner by the archaeal exosome, but short substrates are cleaved distributively [41]. The possibility of quaternary changes in PNPase may also explain observations that ATP, citrate and cyclic di-GMP can modulate the activity of the PNPase [42–45]. The potential of allosteric response to metabolites opens the possibility of a link of RNA turnover with metabolic status as another function of PNPase.

The interaction of PNPase with RNase E plays an important role in the formation of an RNA–degradosome complex in bacteria. The interaction of *E. coli* PNPase with RNase E has been the subject of several previous studies, and indeed the structural basis of this interaction was highlighted by the crystal structure of the complex [23], where the PNPase recognition peptide from RNase E was shown to form a strand as part of a continuous sheet of PNPase. The interaction between PNPase and the RNase E recognition motif in *C. crescentus* is markedly different from that seen in *E. coli.* In our *C. crescentus* structure, it is clear that the GWW motif is buried into a hydrophobic pocket on the surface of PNPase, at a site approximately 60 Å from the position corresponding to the RNase E recognition site in *E. coli*. This is a fascinating example of convergent evolution, where different bacterial classes have arrived at distinct means of recruiting PNPase into a complex with RNase E.

The structural data presented here reveal how the versatile KH fold can be used for RNA recognition and translocation, achieved through the spatial arrangement and cooperative interplay between independent KH domains. The conformational adjustments we observe in PNPase associated with RNA binding provide further insight into the mechanism of this fascinating molecular machine.

## Supplementary Material

supplementary figures

## Supplementary Material

supplementary figure legends
