# Supplementary material for: Crystal structure of Caulobacter crescentus polynucleotide phosphorylase reveals a mechanism of RNA substrate channelling and RNA degradosome assembly
Source: Open Biol. 2012 Apr;2(4):120028. doi: 10.1098/rsob.120028 (PMC3376730; doi:10.1098/rsob.120028)
Supplement: supplementary figures [file rsob120028-s1.pdf]

Figure S1

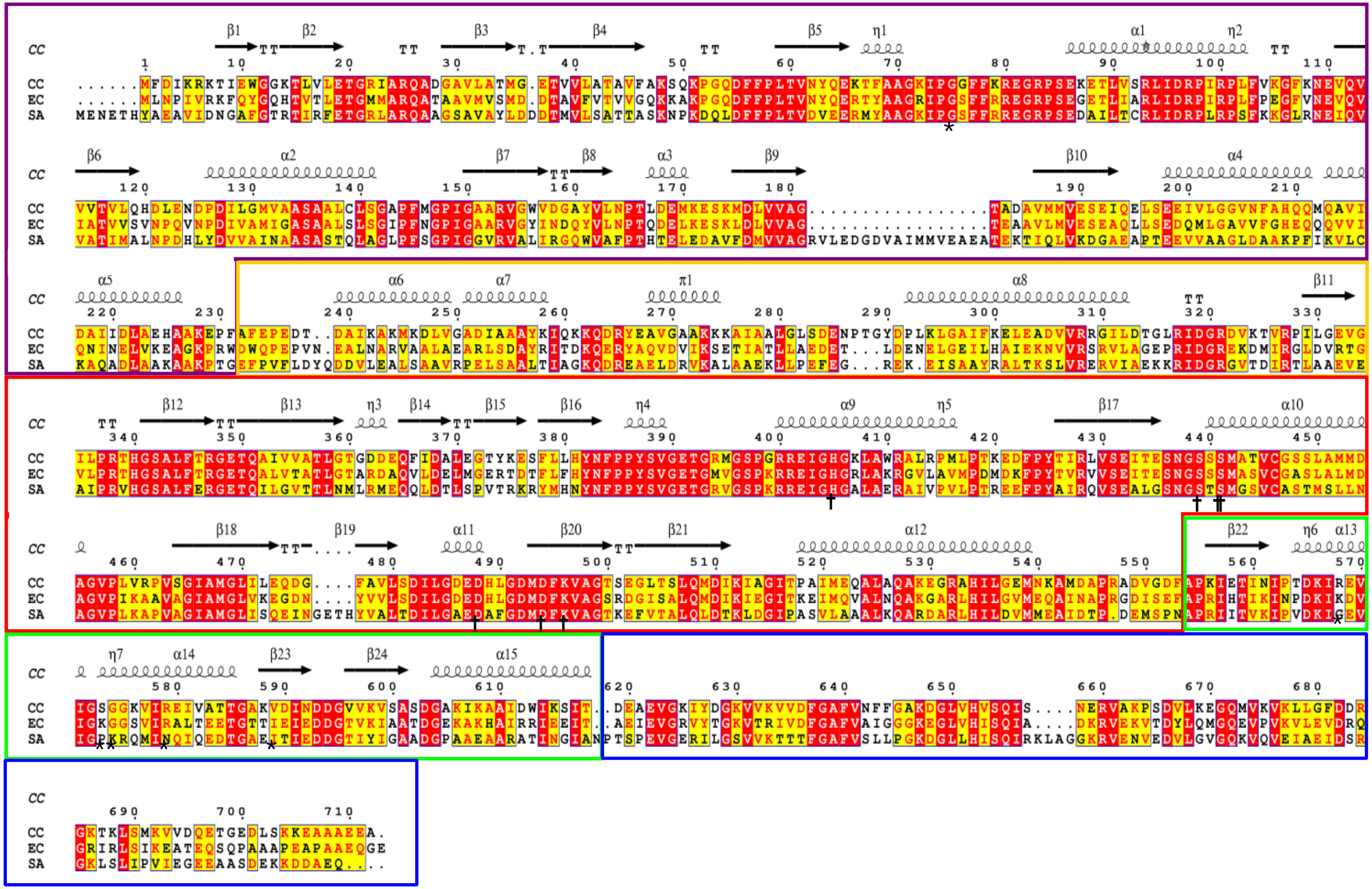

Figure S2

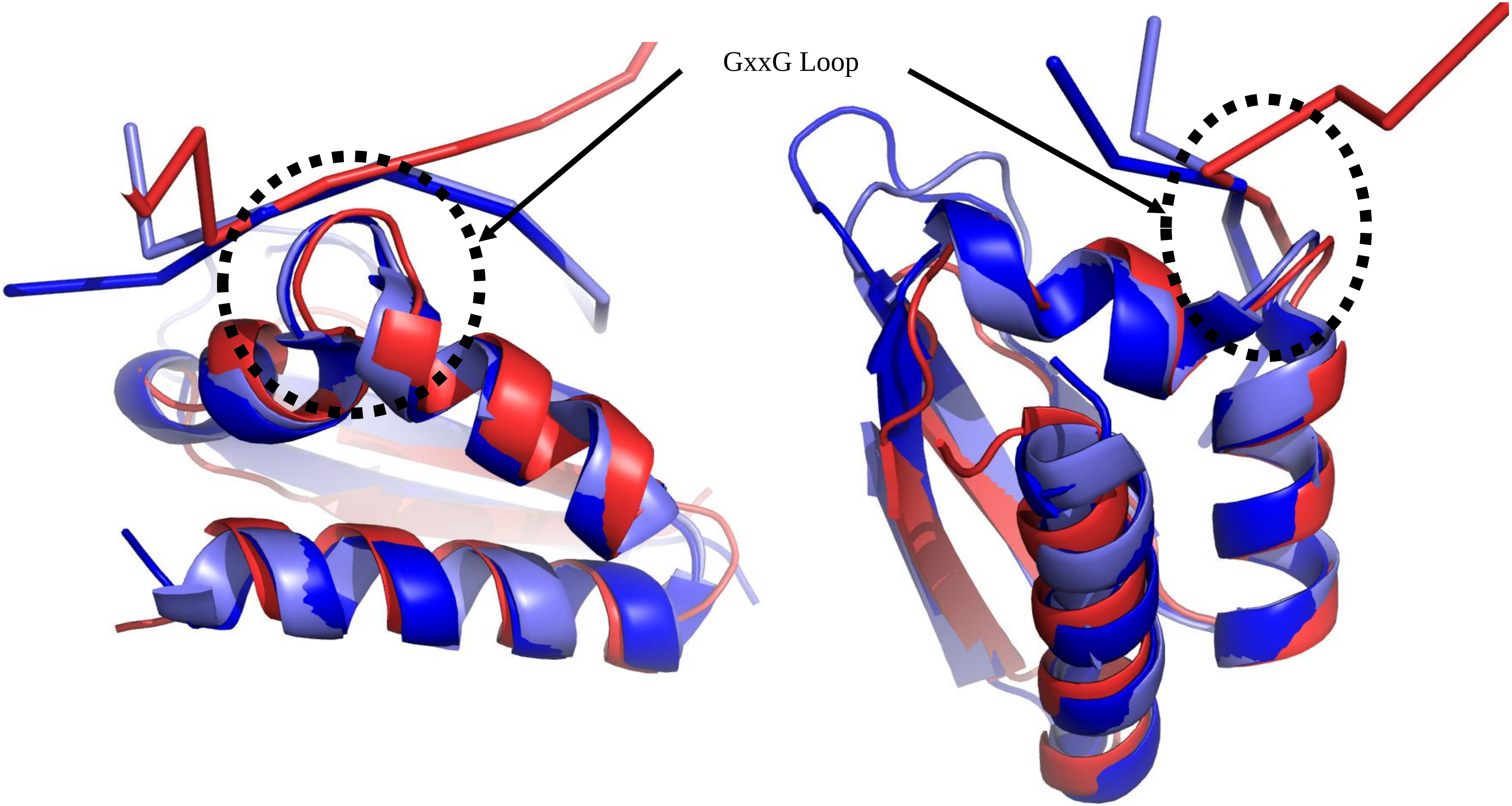

Figure S3

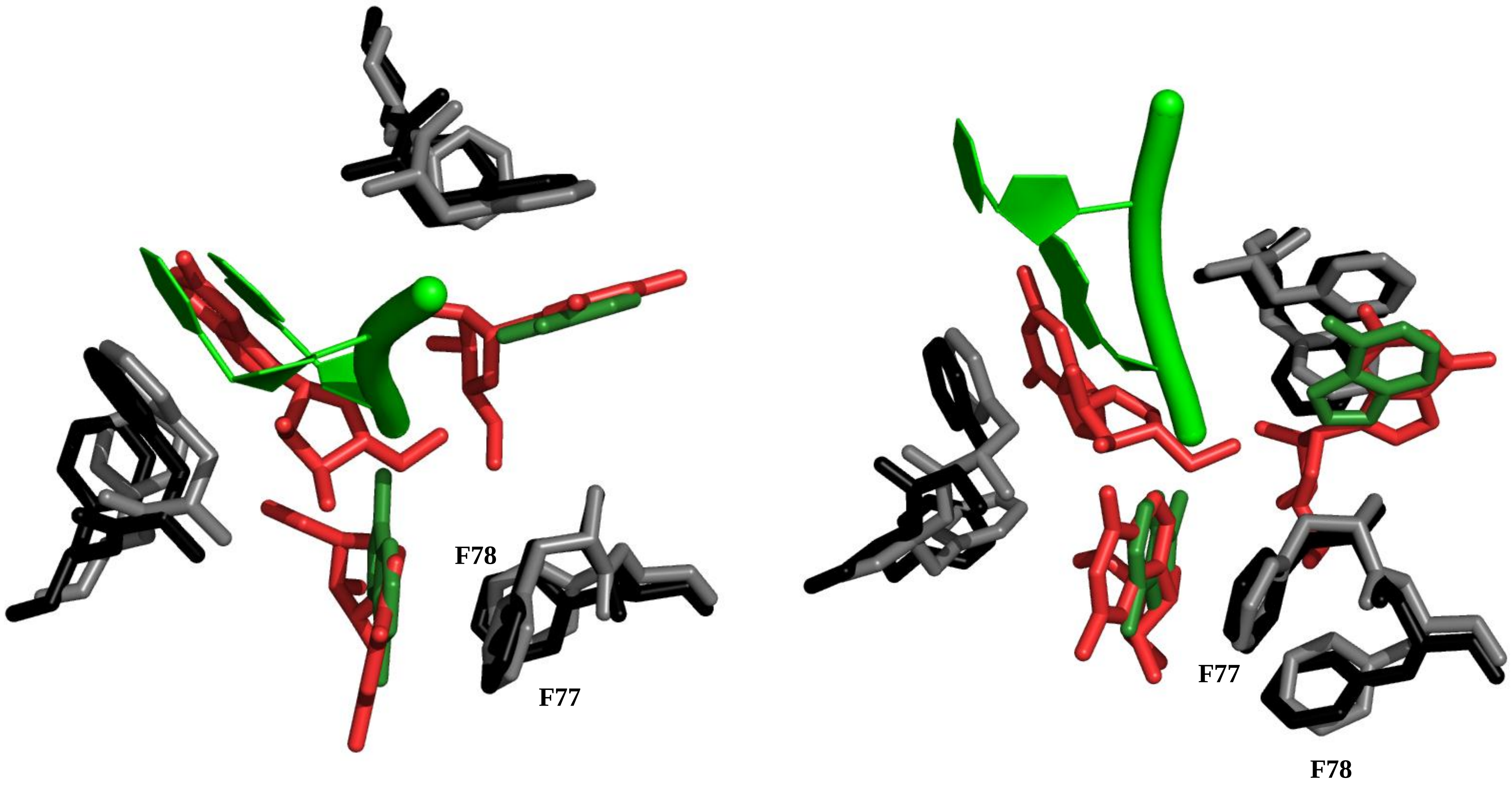

Figure S4

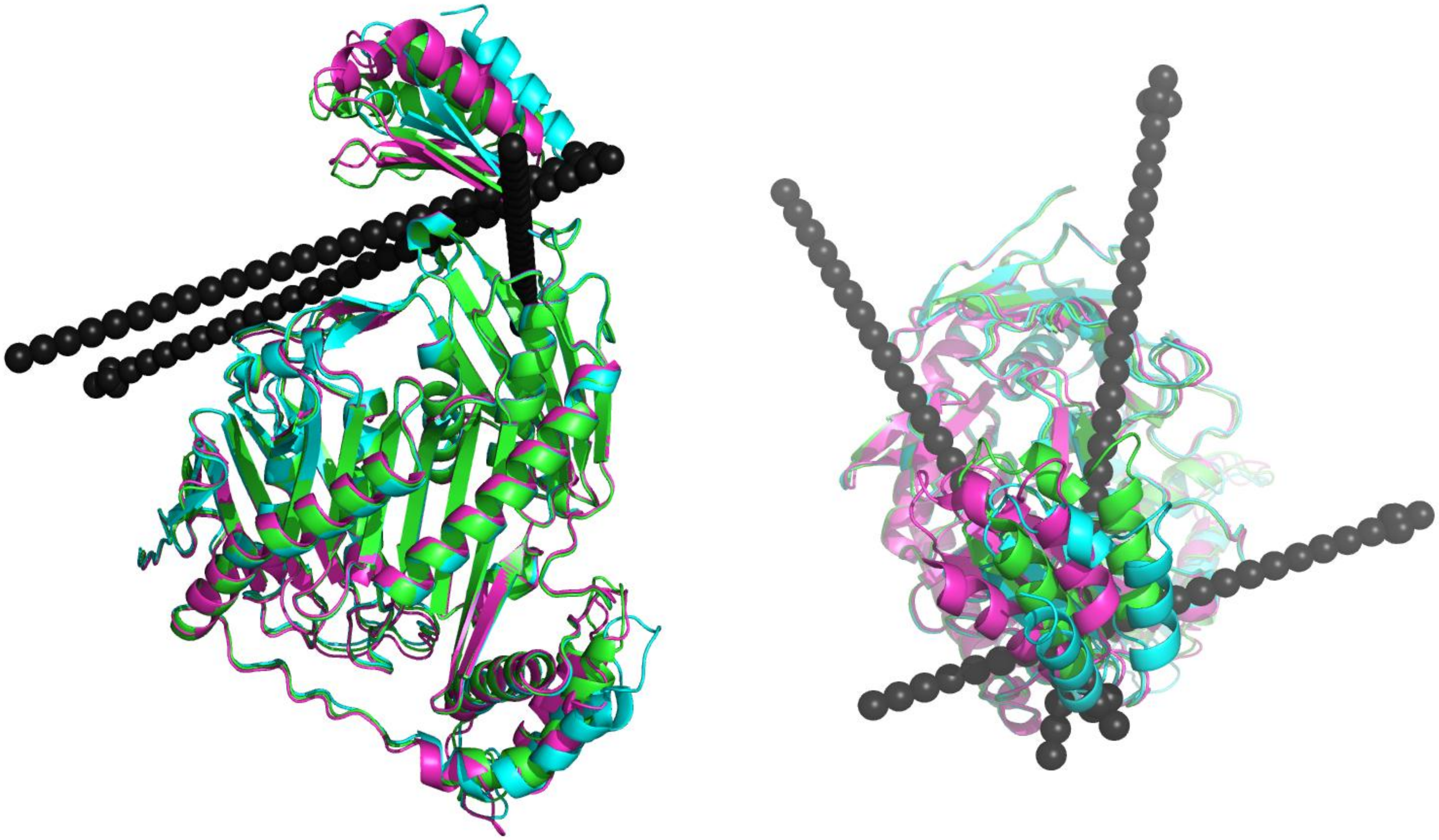

Figure S5

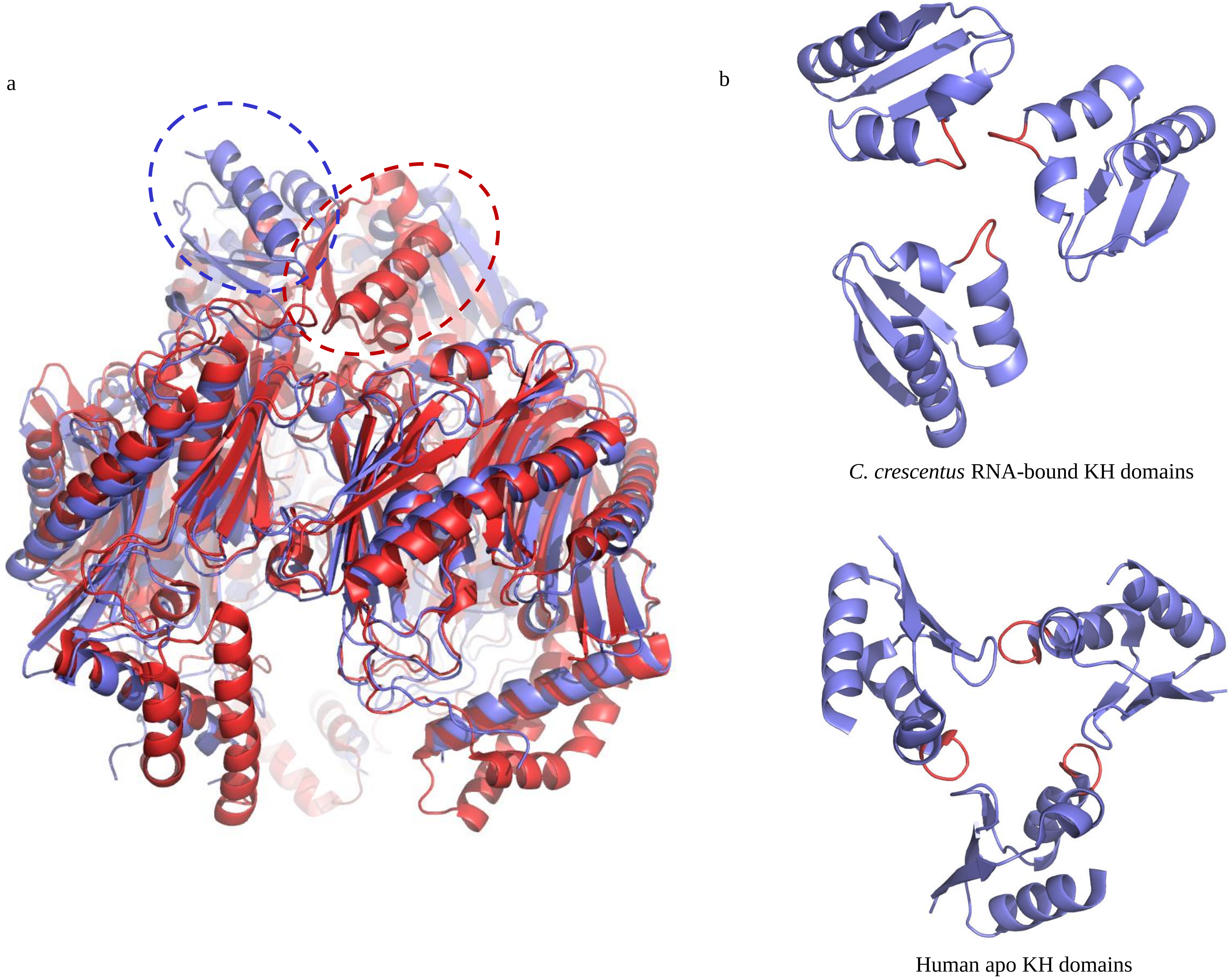

Figure S6

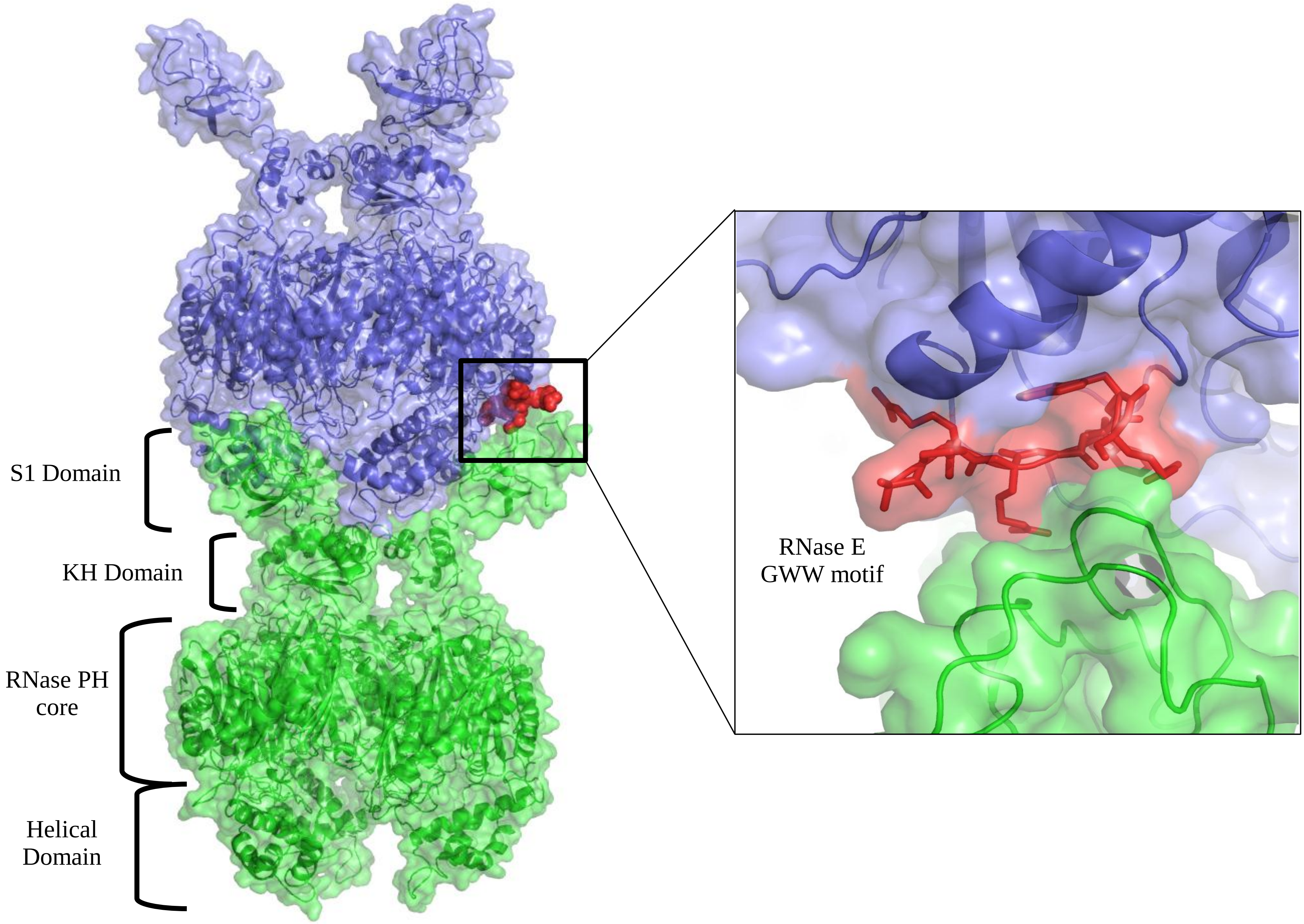
S1 Domain
KH Domain
RNase PH
core
Helical
Domain
RNase E
GWW motif
