# Supplementary material for: Crystal structure of Caulobacter crescentus polynucleotide phosphorylase reveals a mechanism of RNA substrate channelling and RNA degradosome assembly
Source: Open Biol. 2012 Apr;2(4):120028. doi: 10.1098/rsob.120028 (PMC3376730; doi:10.1098/rsob.120028)
Supplement: supplementary figure legends [file rsob120028-s2.doc]

Supplementary Figure 1. Structure based sequence alignment of *C. crescentus* (CC), *E. coli* (EC) and *Streptomyces antibioticus* (SA) PNPase. The individual structural subdomains are in boxes coloured using the same scheme as Figure 1A. Secondary structural elements are indicated above the alignment (β-beta sheet, α-alpha helix, π-pi helix, η-3_10_ helix). Crosses (†) mark key residues of the catalytic site, involved in binding phosphate or metal co-factor. The residues in the KH domain indicated by a star are observed to contact the RNA bases or backbone in the *C. crescentus* PNPase. Alignments were made using ESPript (<http://espript.ibcp.fr/ESPript>).

Supplementary Figure 2. The type I KH-domain of PNPase. Two views of a single KH domain of *C. crescentus* PNPase bound to RNA (red) superposed on the type I KH-domains of human poly(C) binding protein bound to DNA (dark blue; PDB entry 2P2R), and RNA (light blue; 2PY9). The KH domains are shown in cartoon representation and nucleic acid is shown as string. The conserved GxxG nucleic acid binding loops are highlighted by dashed circles.

Supplementary Figure 3. RNA stacking on the FF loops. The overlay of the *C. crescentus* (grey) and *E. coli* (black; PDB 3GCM) FF loops with bound RNA. The main RNA chain bound to the *C. crescentus* PNPase is shown as a light green cartoon, and two bases in partial occupancy are shown in dark green, whilst RNA bound to the crystal structure of the *E. coli* PNPase catalytic core (lacking the S1 and KH domains) is shown in red. The overlay shows that the stacking interaction is similar in both enzymes. The left panel is the entrance to the core viewed along the trimer 3-fold axis, and the right panel is a tilted view.

Supplementary Figure 4. KH domain rotation axes. The three protomers of PNPase from the RNA bound structure are overlayed and coloured in blue, green and magenta. The rotation axes to generate the transformation between the three KH domain orientations are represented as black spheres (calculated using Dyndom). The left panel is a view with the molecular three-fold axis aligned vertically, and the right panel is a view along the three-fold axis.

Supplementary Figure 5. Comparison of the *C. crescentus* and human PNPase structures. (a) Structural overlay of the *C. crescentus* PNPase trimer (blue) and human PNPase trimer (red), revealing marked differences in the orientation of the respective KH domains (indicated by dashed ovals). (b) Comparison of the KH apertures between the two structures. Top; *C. crescentus* PNPase KH domains (blue) with the RNA binding GxxG loops (red) positioned to cooperatively engage RNA. Bottom; Human PNPase KH domains and GxxG loops (coloured as above).

Supplementary Figure 6. The GWW peptide bridges neighbouring PNPase trimers in the crystal. Two trimers of PNPase seen in the P6_3_ crystal lattice are shown as blue and green semi-transparent surfaces. The GWW peptide is shown as a red surface bridging these two molecules. The boxed area shows a zoomed view of this interaction.

Supplementary Movie 1. Rotary like movement of the KH domains. The PNPase trimer (protomers shown as cartoons coloured red, green and blue) is viewed looking down the molecular three-fold axis, with the KH domains in the fore ground. The KH domains morph between the three orientations seen in the RNA bound crystal structure.

Supplementary Movie 2. Quaternary changes in the catalytic core of PNPase. The PNPase trimer (with KH and helical domains removed for clarity) is viewed with the molecular three-fold axis running from top to bottom. The protomers are shown as red, blue and green cylinder representations, and morph between the three states seen for the core region of PNPase in the RNA bound structure.
